# An LC-MS/MS method for the simultaneous determination of 18 antibacterial drugs in human plasma and its application in therapeutic drug monitoring

**DOI:** 10.3389/fphar.2022.1044234

**Published:** 2022-11-08

**Authors:** Wei Lu, Meng Pan, Hongqin Ke, Jun Liang, Wenbin Liang, Ping Yu, Penghua Zhang, Qibin Wang

**Affiliations:** ^1^ Department of Pharmacy, Taihe Hospital, Hubei University of Medicine, Shiyan, Hubei, China; ^2^ College of Pharmacy, Hubei University of Medicine, Shiyan, Hubei, China; ^3^ Department of Cardiovascular Medicine, Taihe Hospital, Hubei University of Medicine, Shiyan, Hubei, China

**Keywords:** antibacterial drugs, pharmacokinetics, pharmacodynamics, UPLC-MS, therapeutic drug monitoring

## Abstract

Antimicrobial resistance (AMR) is a major threat to global health due to the wide use of antibacterial drugs. Multiple studies show that the pharmacokinetic/pharmacodynamic (PK/PD) studies of antibiotics are an approach to prevent/delay AMR. The pharmacokinetic parameters of antibiotics are the basis of PK/PD studies, and therapeutic drug monitoring (TDM) is the key method to obtain pharmacokinetic information. We developed an ultra-performance liquid chromatography–tandem mass spectrometry to determine 18 antibacterial drugs (piperacillin, cefazolin, cefuroxime, cefoperazone, ceftriaxone, cefepime, aztreonam, meropenem, imipenem, levofloxacin, moxifloxacin, azithromycin, clindamycin, tigecycline, linezolid, vancomycin, voriconazole and caspofungin) in human plasma for practical clinical usage. Samples were prepared using protein precipitation with methanol. Chromatographic separation was accomplished in 6 min on a BEH C_18_ column (2.1 × 100 mm, 1.7 µm) using a gradient elution of acetonitrile and 0.1% formic acid in water at a flow rate of 0.3 ml/min. The electrospray ionization source interface was operated in the positive and negative ionization modes. Inter- and intra-day precision, accuracy, recovery, matrix effect, and stability were validated according to the Food and Drug Administration guidance. The correlation coefficients of calibration curves were all greater than 0.99. The accuracies of the 18 antibacterial drugs ranged from 89.1% to 112.4%. The intra-day precision of the analytes ranged from 1.4% to 9.3% and the inter-day precision from 2.1% to 7.2%. The matrix effects ranged from 93.1% to 105.8% and the extraction recoveries ranged between 90.1% and 109.2%. The stabilities of the 18 antibacterial drugs in plasma were evaluated by analyzing three different concentrations following storage at three storage conditions. All samples displayed variations less than 15.0%. The validated method was successfully applied to routine clinical TDM for 231 samples.

## 1 Introduction

Antibacterial drugs are the most important and commonly used therapeutic drugs that are widely used in a clinical setting to treat various diseases, especially infectious diseases. According to the World Health Organization (WHO), the global per-capita antibiotic consumption increased by 39% between 2000 and 2015, and the per-capita consumption showed a steady, rapid growth (Klein et al., 2021). However, with the increased use of antibacterial drugs, the increase in resistance against multiple currently available antibiotics has led to a rapid loss of drug efficacy and a lack of treatment options to treat infectious diseases. Thus, antimicrobial resistance (AMR) poses a global problem (Huemer et al., 2020; Aslam et al., 2021.). The WHO and many governments are taking various approaches to increase the understanding of AMR, promote the rational use of antibacterial drugs, and prevent the emergence of AMR (Nellums et al., 2018; Rochford et al., 2018). As AMR results partially due to the misuse or abuse of antibiotics, rational antibacterial use can help prevent this situation (Moreheadn and Scarbrough, 2018).

Pharmacokinetic/pharmacodynamic (PK/PD) studies have been proven as effective methods to understand the rational use of antibacterial drugs (Scaglione and Paraboni, 2008; Sinnollareddy et al., 2012; Veiga and Paiva, 2018). The clinical effects are conditioned by complex interactions among the three elements of antibiotic therapy, namely, the host, the microorganism, and the drug (Couet, 2018). PK/PD studies focus on the combination of drug concentration with time and antibacterial effect to clarify the mechanism of antibacterial or bactericidal effect at blood or tissue concentrations at specific doses or by administration scheme (Asin et al., 2015). Therefore, PK/PD studies help optimize antibacterial dosing regimens to increase drug efficacy and avoid adverse reactions and AMR.

In the PK/PD study, three indicators were used as reference: concentration of the drug over the minimum inhibitory concentration (MIC) (T > MIC), peak concentration: MIC ratio (C_max_/MIC), and the 24-h area under the concentration (AUC)-time curve divided by the MIC (AUC/MIC) (Mouton et al., 2005; Rodriguez et al., 2021). The PK/PD indices are varying in different kinds of antibacterial drugs, even the targets of the same drug are different when they treat with different bacterias. Lepak and Andes. (2014), Williams et al. (2019), Abdul-Aziz et al. (2020). Thus, the pharmacokinetic parameters of antimicrobial drugs (C_max_ and AUC) form the basis of PK/PD studies. Obtaining pharmacokinetic information on antimicrobial drugs rapidly and accurately is very important. Therapeutic drug monitoring is a clinical practice of measuring specific drugs concentration in a patient’s body fluid (blood, urine, and saliva), elucidate the relationship between drug concentrations and drug effects (pharmacokinetic parameters) based on pharmacokinetic and pharmacokinetic principles (Kang and Lee, 2009). TDM can help us to obtain the pharmacokinetic information. At present, the chromatographic methods and immunoassays are the main detection methods of TDM (Ates et al., 2020).

Although there are numerous studies on the determination of the concentration of antibacterial drugs using liquid chromatography-mass spectrometry (LC-MS) (Parker et al., 2017; Luo et al., 2020; Rehm and Rentsch, 2020a; Rehm and Rentsch, 2020b; Ferrari et al., 2019), most have focused on one class (β-lactam, antifungals, glycopeptides, etc.) or several antibiotics, and there are only a few studies reporting the simultaneous determination of multiple antimicrobial drugs. A combination of antimicrobial drugs has always been used to treat critically ill individuals, children, and the elderly. Drug pharmacokinetics vary among these patient groups (Hahn et al., 2017), thus warranting their study. Therefore, we established and validated a high-throughput ultra-performance liquid chromatography–tandem mass spectrometry (UPLC-MS/MS) method and analyzed human plasma for the quantification of 18 antibacterial drugs (including penicillins, cephalosporins, carbapenems, monobactams, quinolones, macrolides, tetracycline, oxazolidinones, glycopeptides, antifungal, etc.) were commonly used in clinical setting by using LC-MS/MS to facilitate TDM.

## 2 Materials and methods

### 2.1 Chemicals and reagents

**FIGURE 1:**
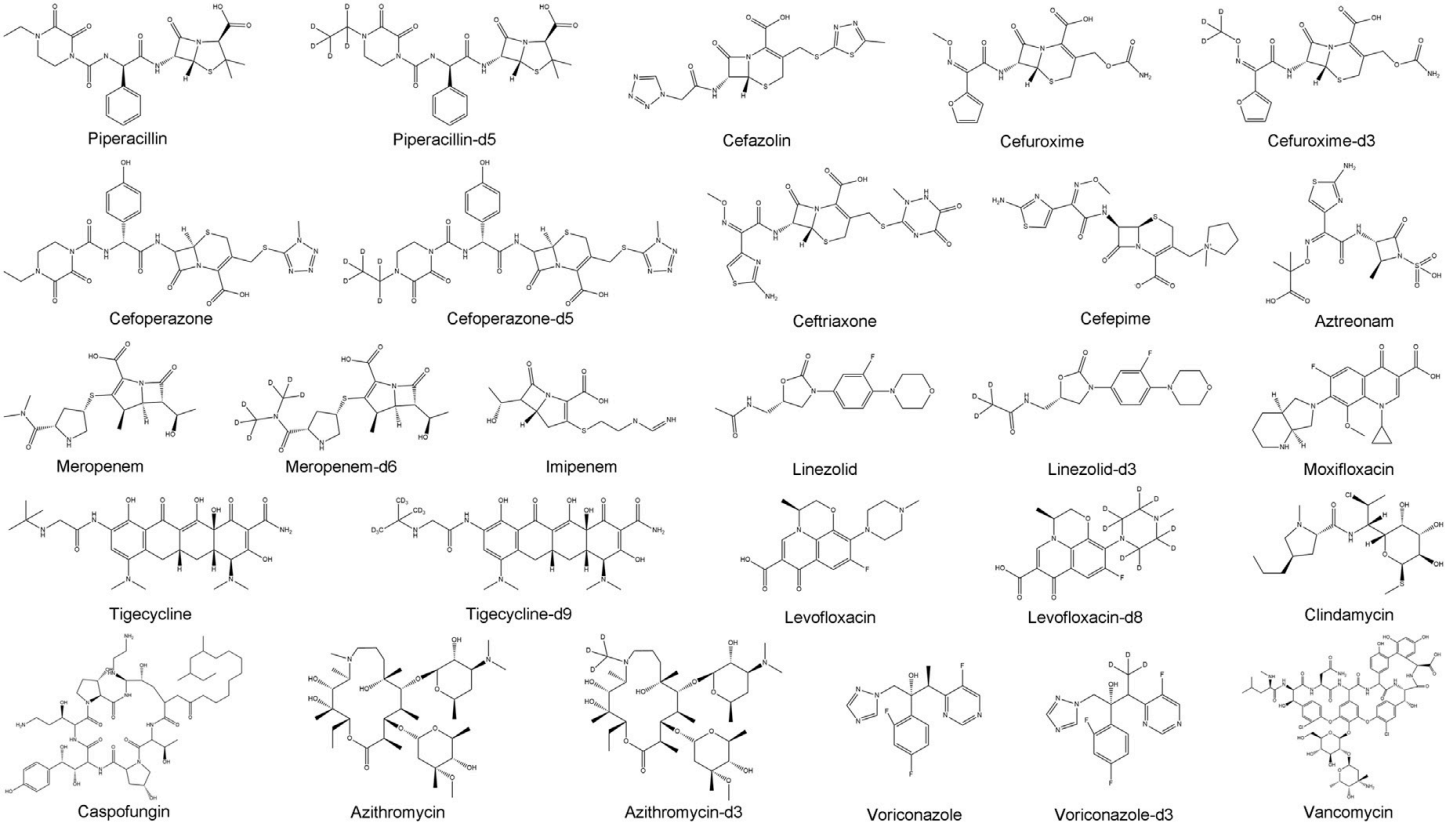
Chemical structures of 18 antimicrobial agents and nine internal standards.

Piperacillin, cefazolin, cefuroxime, cefoperazone, ceftriaxone, cefepime, aztreonam, meropenem, imipenem, levofloxacin, moxifloxacin, tigecycline, linezolid, azithromycin, clindamycin, voriconazole, caspofungin, vancomycin, piperacillin-d5, cefuroxime-d3, cefoperazone-d5, meropenem-d6, levofloxacin-d8, tigecycline-d9, azithromycin-d3, linezolid-d3 and voriconazole-d3 (Figure 1) were purchased from the National Institutes for Food and Drug Control (Beijing, China), Shanghai Yuanye Bio-Technology Co., Ltd. (Shanghai, China) and Shanghai, ZZBIO Co., Ltd. (Shanghai, China). Formic acid was provided by Sigma-Aldrich Co. (Missouri, United States). HPLC grade acetonitrile and methanol were purchased from Merck (Darmstadt, Germany). Redistilled and deionized water was used throughout the study. The blank plasma collected from volunteers or clinical patients who did not used these antibacterial.

### 2.2 Instrumentation and LC-MS/MS conditions

The analysis was performed using a Waters ACQUITY UPLC system (Waters, Milford, MA, United States) and a Micromass Quattro Micro API mass spectrometer (Waters, Milford, MA, United States). The electrospray ionization (ESI) source interface was operated in the positive and negative ionization modes in our study. The following parameters were used: capillary voltage: 3.1 kV, source temperature: 150°C, desolvation temperature: 400°C. Nitrogen was used as the desolvation and cone gas at a flow rate of 800 L h^−1^ and 50 L h^−1^, respectively. Argon was used as collision gas at a flow rate of 0.17 ml min^−1^ in the collision cell. Collision energies and cone voltages were optimized for each analyte individually. The MS/MS parameters for antibacterial drugs and ISs are shown in Table 1.

**TABLE 1:** Optimized multiple reaction monitoring parameters for 18 antimicrobial agents and nine ISs.

Compounds	MRM transitions (m/z)	Cone energy (V)	Collision energy (V)	ESI
Piperacillin	518.3/143.2	16	18	ES+
Cefazolin	455.0/323.3	26	24	ES+
Cefuroxime	423.2/318.1	14	8	ES-
Cefoperazone	646.5/143.1	18	34	ES+
Ceftriaxone	555.2/396.1	30	24	ES+
Cefepime	481.2/396.2	20	16	ES+
Aztreonam	434.14/96.0	30	20	ES-
Meropenem	384.1/141.1	25	18	ES+
Imipenem	300.2/141.9	35	28	ES+
Levofloxacin	362.1/318.2	30	18	ES+
Moxifloxacin	402.3/384.3	30	20	ES+
Tigecycline	586.3/513.4	30	28	ES+
Azithromycin	749.6/591.6	40	38	ES+
Linezolid	338.3/295.8	30	18	ES+
Clindamycin	425.3/126.2	32	28	ES+
Voriconazole	350.3/281.1	20	34	ES+
Caspofungin	547.6/137.2	25	20	ES+
Vancomycin	725.6/144.2	25	13	ES+
Piperacillin-d5	523.3/148.3	18	18	ES+
Cefuroxime-d3	426.4/321.3	15	10	ES-
Cefoperazone-d5	651.2/148.3	20	30	ES+
Meropenem-d6	390.2/147.3	28	20	ES+
Levofloxacin-d8	370.1/326.1	32	18	ES+
Tigecycline-d9	595.1/514.0	35	28	ES+
Azithromycin-d3	752.4/594.3	45	38	ES+
Linezolid-d3	341.1/296.1	31	23	ES+
Voriconazole-d3	353.2/284.1	20	34	ES+

Chromatographic separation was performed on an ACQUITY UPLC^®^ BEH C_18_ Column (2.1 × 100 mm; 1.7 µm). The mobile phase consisted of 0.1% formic acid in water (solvent A) and 0.1% formic acid in acetonitrile (solvent B) used at a flow rate of 0.3 ml min^−1^. The gradient elution program was as follows: 0–0.5 min, 10% B; 0.5–1.2 min, 10%–35% B; 1.2–3.5 min, 35%–70% B; 3.5–4.2 min, 70%–90% B; 4.2–5.2 min, 90% B; 5.2–5.5 min, 90%–10% B; and 5.5–6.0 min, 10% B. The column was maintained at 45°C, the autosampler was set at 4°C. and the injection volume was 5 µL.

### 2.3. Preparation of standard and quality control samples

Based on the solubility of the antibacterial drug, stock solutions of the antimicrobial agents were prepared in MeOH: DMSO (v/v = 1:1). Internal standards (ISs) were prepared in methanol. Mixed working solutions of antibacterial drugs and ISs were prepared in methanol by diluting the stock solutions. Mixed QC solution and calibration solution of antibacterial drugs (50 µL) was added to centrifuge tubes and evaporated under nitrogen, respectively. Next, 50 µL blank plasma was added and mixed by vertexing for 5 min to prepare the QC samples and samples of calibration curve. Whole blood QC samples were prepared in a similar method with blank blood. All calibration and QC samples were freshly prepared before analysis. The concentrations of calibrators and QCs are summarized in Table 2. The concentrations of piperacillin-d5, cefuroxime-d3, cefoperazone-d5, meropenem-d6, levofloxacin-d8, tigecycline-d9, azithromycin-d3, linezolid-d3, and voriconazole-d3 were 18.92 μg mL^−1^, 20.21 μg mL^−1^, 21.05 μg mL^−1^, 18.97 μg mL^−1^, 3.79 μg mL^−1^, 0.45 μg mL^−1^, 2.24 μg mL^−1^, 4.35 μg mL^−1^, and 1.54  μg mL^−1^, respectively.

**TABLE 2:** Concentrations of calibrators and QCs (µg·mL^−1^).

Compounds	Calibration concentrations							QC concentrations				Diluting samples
	1	2	3	4	5	6	7	LLOQ	Low	Medium	High	
Piperacillin	2.18	4.36	10.89	21.78	43.56	108.90	217.80	2.18	6.97	34.85	174.24	435.60
Cefazolin	2.14	4.28	10.70	21.40	42.80	107.00	214.00	2.14	6.85	34.24	171.20	428.00
Cefuroxime	2.16	4.32	10.80	21.60	43.20	108.00	216.00	2.16	6.91	34.56	172.80	432.00
Cefoperazone	2.40	4.80	12.00	24.00	48.00	120.00	240.00	2.40	7.68	38.40	192.00	480.00
Ceftriaxone	2.20	4.40	11.00	22.00	44.00	110.00	220.00	2.20	7.04	35.20	176.00	440.00
Cefepime	2.22	4.44	11.10	22.20	44.40	111.00	222.00	2.22	7.10	35.52	177.60	444.00
Aztreonam	2.15	4.29	10.73	21.45	42.90	107.25	214.50	2.15	6.86	34.32	171.60	429.00
Meropenem	2.02	4.04	10.10	20.20	40.40	101.00	202.00	2.02	6.46	32.32	161.60	404.00
Imipenem	1.99	3.98	9.95	19.90	39.80	99.50	199.00	1.99	6.37	31.84	159.20	398.00
Levofloxacin	0.55	1.10	2.76	5.51	11.03	27.56	55.13	0.55	1.76	8.82	44.10	110.25
Moxifloxacin	0.28	0.57	1.42	2.84	5.69	14.22	28.44	0.28	0.91	4.55	22.75	56.88
Tigecycline	0.06	0.12	0.29	0.58	1.15	2.88	5.76	0.06	0.18	0.92	4.61	11.52
Azithromycin	0.22	0.43	1.09	2.17	4.34	10.85	21.70	0.22	0.69	3.47	17.36	43.40
Linezolid	0.39	0.77	1.93	3.85	7.70	19.25	38.50	0.39	1.23	6.16	30.80	77.00
Clindamycin	0.22	0.43	1.08	2.16	4.31	10.78	21.56	0.22	0.69	3.45	17.25	43.12
Voriconazole	0.10	0.20	0.51	1.02	2.04	5.10	10.20	0.10	0.33	1.63	8.16	20.40
Caspofungin	0.12	0.24	0.60	1.20	2.40	6.00	12.00	0.12	0.38	1.92	9.60	24.00
Vancomycin	0.41	0.81	2.03	4.06	8.11	20.28	40.55	0.41	1.30	6.49	32.44	81.10

### 2.4 Sample preparation

Two sample preparation methods were used in our study. For tigecycline and caspofungin, 50 µL sample plasma and 50 µL ISs solution were mixed and 400 µL methanol was added. The mixture was vortex-mixed for 60 s to precipitate proteins. After centrifugation at 20,800 *g* for 10 min (at 4°C), the supernatant was transferred for sampling analysis. For other antibacterial drugs (except tigecycline and caspofungin), 50 µL sample plasma and 50 µL ISs solution were mixed, and then 400 µL methanol was added. The mixture was vortex-mixed for 60s to precipitate proteins. After centrifugation at 20,800 *g* for 10 min (at 4°C), the supernatant (100 µL) was transferred to a new tube and 900 µL water (0.1% formic acid) was added. After vortex-mixing for 60 s, the mixture was transferred for analysis.

### 2.5 Method validation

The methods were following the principles of the bioanalytical method validation guideline (FDA, 2018; Chinese Pharmacopoeia Commission, 2020), and other articles (Matuszewski et al., 2003; Patel et al., 2016; Zhang et al., 2018). The method validation included selectivity, specificity, cross talk, carryover, calibration curve, matrix effects, extraction recovery, precision and accuracy, stability, and dilution effects.

#### 2.5.1 Selectivity, specificity, cross talk, and carryover

The selectivity and specificity of the method were evaluated by monitoring and comparing the quantification ions of the antibacterial drugs and ISs in blank human plasma from six different sources with those in blank human plasma spiked with analytes at the lower limit of quantification (LLOQ) to check for possible interference. And the blank plasmas were collected from clinical patients, including normal heparinized, hemolysis, hyperlipidemia, and hyperbilirubinemia plasma samples. Cross talk phenomena among MS/MS channels were assessed by injecting the 18 antimicrobials and nine labeled ISs single working solutions and monitoring the response in the other MS/MS channels. Carryover was assessed by comparing an extract of blank plasma injected immediately after the highest calibration standard injected in triplicate. The blank matrix should demonstrate no significant response at the retention times of the antibacterial drugs and ISs.

#### 2.5.2 Linearity of the calibration curve and LLOQ

Linearity was evaluated by analyzing calibration curves using seven concentration points. Calibration curves were constructed by plotting peak area ratios (analyte/internal standard) versus plasma concentrations. Linear weighted least-squares analysis was performed, and a weighting factor of 1/x^2^ was used. A coefficient of determination (r^2^) > 0.99 was expected in all calibration curves. The lowest calibration points in the calibration curve were considered as LLOQ.

#### 2.5.3 Matrix effects and extraction recovery

The following three different sets of solutions were prepared at LQC, MQC, and HQC level (A) blank plasma sample spiked with analytes and IS before extraction (B) blank plasma sample spiked with analytes and IS after extraction, and (C) water as a substrate spiked with analytes and ISs for sample extraction. Matrix effects were determined from the ratio of peak areas from the post-extraction spiked serum and pure water substrate (B)/(C). Extraction recovery was determined from the ratio of peak areas from pre-extraction and post-extraction spiked sera (A)/(B). All matrix effects and extraction recoveries were determined at three concentrations, and the QC samples were prepared using one source of plasma. The ratio of extraction recoveries should be >85% and <115%, whereas the coefficients of variation (CV, %) should be <15%.

#### 2.5.4 Precision and accuracy

Intra-day precision and accuracy were evaluated in six replicates at three QC levels (LLOQ, low, medium, and high concentrations) within 1 day during the same analytical run. Inter-day precision and accuracy were assessed based on the analysis of the same QC samples on three consecutive days. RSD was evaluated to determine precision, and accuracy was represented by a percentage of the nominal concentration (%). The precision and accuracy should be within 15% for the three QC levels.

#### 2.5.5 Stability

The stability of antibacterial drugs was determined by analyzing three level concentration of QC samples stored under four different storage conditions. Freeze-thaw stability was determined after three freeze-thaw cycles (from −20°C to 25°C) on consecutive days. Long-term stability was studied by storing QC samples at −80°C for 14 days and short-term stability was determined by analyzing QC samples stored at 25°C for 6 h. Post-processing stability was evaluated after 24 h of storage in the sample manager at 4°C.The blood sample stability were also evaluated by the blood QC samples stored at 25°C for 6 h. Analyte concentrations were compared with those of freshly prepared QC samples and were considered stable if the accuracy and precision were within ± 15%.

#### 2.5.6 Dilution effects

To verify dilution effects, blank plasma was spiked in stock solutions to make diluting QC samples, the concentration of diluting QC samples is summarized in Table 2. Subsequently, these high concentrated plasma samples were diluted 5-fold with blank plasma (*n* = 6) before extraction and analyzed with calibration standards prepared on the same day. Accuracy and precision within±15% were set as acceptance criteria.

### 2.6 Applicability of the method for routine TDM

The validated method was used to analyze the steady-state concentrations of antimicrobial agents in plasma samples collected from patients. Samples were collected from patients at least after the 7th dose with the assumption that steady-state plasma levels were attained. Blood samples containing vancomycin and voriconazole were collected at 0.5 h before the next administration, whereas samples containing other drugs were collected 0.5 h after administration. Blood samples were collected in tubes containing heparin sodium as an anticoagulant, centrifuged at 6,000×g for 15 min at room temperature, and immediately stored at –80°C. All samples were analyzed within 4 h of collection. The study protocol was approved by the Ethics Committee of Taihe Hospital, Hubei University of Medicine (Hubei, China), and all patients signed informed consent after they were informed. For routine TDM, a calibration curve was constructed for each batch and QC samples were prepared.

## 3 Results and discussion

### 3.1 Method development and optimization

Method development was commenced by optimizing the ionization and fragmentation conditions for each analyte and IS. The optimization process was achieved by the continuous infusion of each analyte dissolved in methanol/water (50:50, v/v) by mass spectrometry using an internal fluidic pump at a flow rate of 20 μL min^−1^ and concentration of 100–500 ng mL^−1^. Positive and negative ESI modes was selected. After MS/MS optimization, the chromatographic separation conditions were optimized to achieve sufficient separation and symmetrical peak shapes with an adequate response. Several UPLC columns with different modifications of the C_18_ stationary phase were tested, including Acquity UPLC BEH C_18_, Waters CORTECS T_3_, and Waters CORTECS UPLC C_18_. Similarly, a combination of several mobile phases and additives at different concentrations were evaluated. Both methanol and acetonitrile were tested as organic mobile phases. Formic acid at concentrations of 0.05%, 0.1%, and 0.2%, and ammonium formate at concentrations of 2 mM and 5 mM were investigated as additives to the mobile phases. Reasonable retention and resolution were achieved using Acquity UPLC BEH C_18_ with 0.1% formic acid in water and acetonitrile at a flow rate of 0.3 ml min^−1^. A gradient elution program was established with a total run time of 6.0 min. In this study, we developed our LC-MS/MS method for the simultaneous determination of 18 antimicrobial drugs, which covers almost all types of antibacterial drugs. The concurrent use of multiple antibacterial drugs is common in a clinical setting, especially in critically ill patients. Therefore, one of the methods for the simultaneous determination of selected antibacterial drugs is to increase sample throughput and decrease the risk of errors during analysis, such that it can improve patient dependency. Our method was selective and sensitive, which was established by analyzing samples from patients treated with antimicrobial drugs.

### 3.2 Method validation

#### 3.2.1 Selectivity, specificity, cross-talk, and carry-over

**FIGURE 2:**
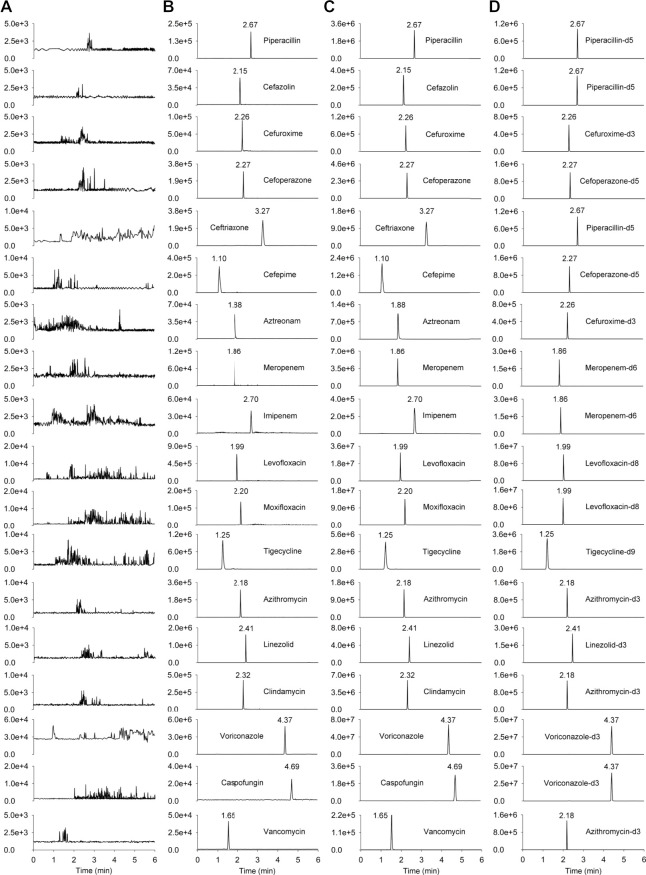
The chromatograms of the 18 antimicrobial agents and ISs. **(A)** blank human plasma sample; **(B)** blank plasma sample spiked with LLOQ; **(C)** patient’s plasma sample collected at 0.5 h after intravenous administration; **(D)** human plasma spiked internal standards (piperacillin-d5 at 18.92 μg mL^−1^, cefuroxime-d3 at 20.21 μg mL^−1^, cefoperazone-d5 at 21.05 μg mL^−1^, meropenem-d6 at 18.97 μg mL^−1^, levofloxacin-d8 at 3.79 μg mL^−1^, tigecycline-d9 at 0.45 μg mL^−1^, azithromycin-d3 at 2.24 μg mL^−1^, linezolid-d3 at 4.35 μg mL^−1^, voriconazole-d3 at 1.54 μg mL^−1^).

Extracted ion chromatograms were compared between the same type of matrix to ensure that there was no interference from endogenous substances or other components. No cross-talk phenomenon was observed among MS/MS channels. Representative chromatograms of blank human plasma, blank plasma sample spiked with LLOQ, and patient’s plasma sample collected at 0.5 h after intravenous administration are shown in Figure 2, the peak area of analytes and ISs in the blank plasma sample injected after the higher limit of quantification sample was <5% of the LLOQ and <1% of the IS, demonstrating that the carry-over effect was negligible.

#### 3.2.2 Linearity and LLOQ

A calibration curve was constructed for each analyte that covered their therapeutic ranges. The LLOQ for each analyte was lower than the therapeutic concentration that corresponded to the expected concentration in patients with poor adherence or nonadherence to medications. The results for the 18 analytes are shown in Table 3. Linearity was achieved with r^2^ > 0.99. The LLOQ was established for each analyte as the lowest point of the calibration curve. The S/N ratio of each LLOQ was >10.

**TABLE 3:** Linear ranges, linear equations, correlation coefficients, and LLOQ of 18 antimicrobial agents.

Compounds	Internal standard	Linear range (µg·mL−1)	Regression equation	r2	LLOQ (µg·mL−1)
Piperacillin	Piperacillin-d5	2.18–217.80	Y = 0.0007 X - 0.0005	0.9970	2.18
Cefazolin	Piperacillin-d5	2.14–214.00	Y = 0.0018X+0.0014	0.9990	2.14
Cefuroxime	Cefuroxime-d3	2.16–216.00	Y = 0.0023X+0.0066	0.9974	2.16
Cefoperazone	Cefoperazone-d5	2.40–240.00	Y = 0.0176X-0.0285	0.9974	2.40
Ceftriaxone	Piperacillin-d5	2.20–220.00	Y = 0.0155X+0.0228	0.9984	2.20
Cefepime	Cefoperazone-d5	2.22–222.00	Y = 0.0042X+0.0074	0.9986	2.22
Aztreonam	Cefuroxime-d3	2.15–214.50	Y = 0.0044X-0.0033	0.9982	2.15
Meropenem	Meropenem-d6	2.02–202.00	Y = 0.0488X+0.0133	0.9994	2.02
Imipenem	Meropenem-d6	1.99–199.00	Y = 0.0306X+0.0446	0.9982	1.99
Levofloxacin	Levofloxacin-d8	0.55–55.13	Y = 0.3319X+0.207	0.9982	0.55
Moxifloxacin	Levofloxacin-d8	0.28–28.44	Y = 0.1868X+0.0036	0.9994	0.28
Tigecycline	Tigecycline-d9	0.06–5.76	Y = 0.1190X+0.0146	0.9986	0.06
Azithromycin	Azithromycin-d3	0.22–21.70	Y = 0.0699X+0.0147	0.9994	0.22
Linezolid	Linezolid-d3	0.39–38.50	Y = 0.4935X+0.0188	0.9981	0.39
Clindamycin	Azithromycin-d3	0.22–21.56	Y = 1.7838X-0.1494	0.9998	0.22
Voriconazole	Voriconazole-d3	0.10–10.20	Y = 1.666X+0.0297	0.9984	0.10
Caspofungin	Voriconazole-d3	0.12–12.00	Y = 0.2094X-0.0018	0.9984	0.12
Vancomycin	Azithromycin-d3	0.41–40.55	Y = 0.0054X-0.0006	0.9980	0.41

#### 3.2.3 Precision and accuracy

The accuracy and intra- and inter-day precisions are shown in Table 4. The accuracies of LLOQ, low, medium, and high QC samples of analytes ranged from 89.1% to 112.6%. The intra- and inter-day precisions of the analytes ranged from 1.4% to 9.7%, which were within acceptable limits. The results demonstrated that the present method was reliable and reproducible for the simultaneous quantification of 18 antimicrobial agents in human plasma.

**TABLE 4:** Intra- and inter-day accuracy and precision of 18 antimicrobial agents in human plasma.

Compounds	Nominal concentration µg·mL−1	Intra-day (%, *n* = 6)		Inter-day (%, *n* = 18)	
		Precision	Accuracy	Precision	Accuracy
Piperacillin	2.18	6.7	109.7	7.9	107.3
	6.97	2.9	112.4	6.6	103.3
	34.85	4.3	99.5	4.7	98.2
	174.24	3.9	92.3	4.8	93.7
Cefazolin	2.14	9.7	105.9	10.3	104.8
	6.85	9.3	108.0	7.2	107.0
	34.24	4.7	101.4	4.3	102.6
	171.20	3.2	98.6	4.1	96.9
Cefuroxime	2.16	5.5	106.2	6.3	106.3
	6.91	3.6	104.4	6.5	102.8
	34.56	4.0	100.7	4.3	103.8
	172.80	4.7	100.3	4.8	98.1
Cefoperazone	2.40	6.2	104.4	8.1	100.8
	7.68	3.6	105.1	5.0	102.9
	38.40	2.5	94.5	3.2	93.7
	192.00	4.8	94.1	5.5	91.1
Ceftriaxone	2.20	4.9	90.0	6.8	93.9
	7.04	3.1	94.4	5.2	92.8
	35.20	1.4	106.2	3.2	106.2
	176.00	5.1	107.3	5.3	102.9
Cefepime	2.22	5.8	112.6	7.2	108.4
	7.10	4.4	108.3	5.3	105.6
	35.52	3.0	108.2	5.5	105.0
	177.60	5.6	100.8	3.4	101.3
Aztreonam	2.15	6.3	105.7	5.3	103.6
	6.86	4.1	99.0	3.4	99.8
	34.32	4.9	92.1	3.9	92.7
	171.60	4.3	91.2	3.4	90.8
Meropenem	2.02	7.7	94.3	5.4	92.2
	6.46	6.5	97.2	5.6	99.1
	32.32	2.0	94.9	3.9	94.2
	161.60	2.5	92.6	2.4	91.1
Imipenem	1.99	6.2	91.9	6.8	90.7
	6.37	4.1	97.7	3.5	98.8
	31.84	2.3	109.0	2.1	108.6
	159.20	5.9	104.5	4.5	103.5
Levofloxacin	0.55	4.3	104.2	5.9	105.1
	1.76	4.0	100.9	4.4	102.1
	8.82	4.8	98.7	5.2	100.4
	44.10	2.9	90.8	3.3	92.5
Moxifloxacin	0.28	5.8	104.2	6.0	103.9
	0.91	3.5	95.4	4.0	98.0
	4.55	5.3	93.3	4.3	96.3
	22.75	3.9	91.1	4.5	93.5
Tigecycline	0.06	5.4	107.1	7.1	110.9
	0.18	5.2	103.4	6.3	106.3
	0.92	2.2	104.1	4.4	102.6
	4.61	5.9	96.8	3.9	97.3
Azithromycin	0.22	5.9	89.9	7.4	93.1
	0.69	2.6	91.3	6.6	96.6
	3.47	2.8	94.7	6.4	100.5
	17.36	2.8	97.0	3.3	98.9
Linezolid	0.39	6.8	105.3	7.0	102.7
	1.23	3.2	96.8	3.8	97.8
	6.16	5.4	94.6	6.0	98.4
	30.80	3.8	93.3	4.3	93.8
Clindamycin	0.22	4.7	105.0	5.6	102.2
	0.69	2.3	103.1	3.8	103.1
	3.45	2.6	92.8	2.7	95.0
	17.25	2.7	89.1	2.8	91.3
Voriconazole	0.10	6.4	106.7	5.2	104.9
	0.33	2.6	103.4	4.4	106.5
	1.63	4.5	105.9	3.5	105.7
	8.16	2.0	105.6	4.1	111.1
Caspofungin	0.12	5.1	103.7	4.9	104.4
	0.38	3.0	100.3	4.3	98.1
	1.92	4.0	95.4	6.1	95.7
	9.60	1.6	91.4	2.8	91.5
Vancomycin	0.41	6.9	108.6	8.0	107.4
	1.30	5.7	111.1	6.8	106.1
	6.49	3.1	102.7	5.0	99.8
	32.44	3.3	94.2	3.3	96.2

#### 3.2.4 Recovery and matrix effect

The matrix effects ranged from 93.1% to 105.5% and the extraction recoveries were between 88.3% and 109.2% for the 18 antimicrobial agents listed in Table 5. All coefficients of variation were <15%. These results demonstrated that pretreatment of plasma samples by protein precipitation could be used to attain stable extraction efficiencies without significant interference from the plasma matrix.

**TABLE 5:** Matrix effects and extraction recoveries of 18 antimicrobial agents in human plasma (mean ± RSD, *n* = 6).

Compounds	Nominal concentration (µg·mL−1)	Extraction recovery (%)	Matrix effect (%)
Piperacillin	6.97	94.7 ± 6.1	100.0 ± 7.7
	34.85	109.2 ± 2.6	95.0 ± 1.9
	174.24	103.9 ± 4.0	104.22 ± 4.9
Cefazolin	6.85	95.3 ± 7.9	97.8 ± 5.3
	34.24	107.7 ± 6.5	101.8 ± 4.7
	171.20	109.0 ± 4.1	99.6 ± 6.0
Cefuroxime	6.91	106.7 ± 5.4	103.2 ± 6.6
	34.56	94.8 ± 5.7	98.4 ± 6.5
	172.80	94.9 ± 7.2	98.8 ± 2.6
Cefoperazone	7.68	107.8 ± 4.9	102.6 ± 6.3
	38.40	95.9 ± 8.9	105.8 ± 6.4
	192.00	102.3 ± 4.2	103.1 ± 3.7
Ceftriaxone	7.04	96.9 ± 5.7	104.3 ± 2.7
	35.20	93.3 ± 7.1	102.6 ± 7.4
	176.00	97.1 ± 3.4	101.7 ± 2.7
Cefepime	7.10	96.4 ± 2.9	103.2 ± 2.9
	35.52	93.7 ± 4.0	100.6 ± 2.0
	177.60	104.1 ± 6.2	96.3 ± 3.7
Aztreonam	6.86	97.3 ± 4.0	100.5 ± 8.8
	34.32	96.1 ± 7.2	96.5 ± 6.7
	171.60	98.6 ± 8.3	102.4 ± 5.3
Meropenem	6.46	91.9 ± 6.3	96.7 ± 3.3
	32.32	92.7 ± 4.2	95.7 ± 4.2
	161.60	96.0 ± 4.4	95.1 ± 3.1
Imipenem	6.37	96.9 ± 4.4	93.1 ± 4.6
	31.84	94.8 ± 3.2	98.2 ± 4.8
	159.20	97.6 ± 4.6	101.4 ± 4.1
Levofloxacin	1.76	103.4 ± 3.6	104.6 ± 6.1
	8.82	93.6 ± 3.7	102.9 ± 6.6
	44.10	98.2 ± 4.1	99.8 ± 6.8
Moxifloxacin	0.91	94.9 ± 8.7	98.8 ± 6.7
	4.55	95.7 ± 5.4	105.5 ± 2.8
	22.75	95.6 ± 6.4	99.0 ± 5.9
Tigecycline	0.18	102.2 ± 3.4	97.8 ± 4.1
	0.92	102.9 ± 3.0	98.4 ± 2.7
	4.61	96.1 ± 7.7	103.4 ± 7.5
Azithromycin	0.69	96.2 ± 3.2	102.9 ± 1.9
	3.47	90.6 ± 1.7	99.1 ± 6.1
	17.36	91.8 ± 4.8	99.1 ± 8.2
Linezolid	1.23	93.6 ± 3.3	98.5 ± 2.7
	6.16	92.0 ± 3.7	99.5 ± 2.5
	30.80	90.1 ± 8.0	96.3 ± 7.2
Clindamycin	0.69	102.7 ± 4.8	102.4 ± 6.4
	3.45	97.8 ± 2.8	102.2 ± 4.6
	17.25	95.9 ± 2.7	98.4 ± 7.6
Voriconazole	0.33	92.7 ± 3.1	105.3 ± 3.7
	1.63	97.7 ± 3.7	105.5 ± 2.0
	8.16	93.9 ± 1.8	103.3 ± 3.6
Caspofungin	0.38	106.8 ± 1.0	95.8 ± 3.8
	1.92	95.7 ± 1.2	94.7 ± 3.9
	9.60	93.3 ± 8.2	105.1 ± 6.1
Vancomycin	1.30	95.9 ± 3.6	100.2 ± 4.7
	6.49	94.4 ± 2.8	98.5 ± 5.4
	32.44	97.6 ± 3.6	97.7 ± 4.2

#### 3.2.5 Stability

The stability of the method is listed in Table 6. The accuracies did not exceed ±12.0%. The CV was within 9.5% for all analytes at room temperature for 6 h, after storage in the sample manager at 4°C for 24 h, and after three freeze-thaw cycles (from −20°C to 25°C). All the analytes were stable in human blood for at least 6 h at 25°C. Although most of the antimicrobial agents could be stably stored for 14 days at −80°C, the accuracy of meropenem, imipenem, vancomycin, and tigecycline were >20% at -80°C for 14 days. Thus, in our study, samples were analyzed within 2 h after preparation and the collected plasma samples were analyzed within 6 h. In addition, no more than three freeze-thaw cycles were performed on plasma samples during storage and transportation.

**TABLE 6:** Stability results of 18 antimicrobial agents in plasma at different storage conditions (% *n* = 6).

Compounds	Concentration (µg·mL−1)	Blood stability		Short-term stability		Post-processing		Freeze-thaw stability		Long-term stability	
		Accuracy	CV	Accuracy	CV	Accuracy	CV	Accuracy	CV	Accuracy	CV
Piperacillin	6.97	96.2	4.8	95.4	2.9	96.3	3.0	93.6	5.5	92.3	7.7
	34.85	94.7	3.6	94.7	3.5	93.7	4.1	94.1	6.0	94.1	8.6
	174.20	92.1	5.2	92.2	2.0	95.4	2.3	91.5	4.4	89.7	5.8
Cefazolin	6.85	95.4	5.3	105.0	3.2	103.5	3.3	97.5	6.4	89.9	7.6
	34.24	92.8	2.8	99.9	4.1	96.4	4.0	95.7	4.5	92.1	8.9
	171.20	94.1	6.2	95.2	3.3	95.0	4.3	91.6	5.2	94.3	10.1
Cefuroxime	6.91	93.4	5.7	104.3	4.9	105.2	5.0	94.6	7.1	93.7	7.9
	34.56	95.1	4.0	99.1	4.1	97.6	4.5	96.5	4.1	92.2	9.2
	172.80	91.9	6.8	96.2	3.0	96.4	5.0	94.4	3.8	90.4	5.6
Cefoperazone	7.68	92.5	4.3	103.2	6.4	96.8	4.3	95.8	5.3	88.6	6.9
	38.40	93.7	4.5	98.4	4.3	95.3	3.8	96.2	5.2	92.1	5.5
	192.00	93.5	4.6	95.2	4.1	94.2	3.0	91.3	4.4	90.5	7.1
Ceftriaxone	7.04	102.5	6.3	105.5	3.5	103.5	3.2	98.6	5.7	93.6	5.2
	35.20	95.7	4.2	107.2	3.0	105.2	3.6	96.4	4.4	95.5	3.3
	176.00	93.6	3.6	95.5	4.0	96.8	4.2	94.4	5.1	90.4	3.9
Cefepime	7.10	92.9	4.3	97.1	5.0	102.3	3.9	98.4	4.3	87.9	7.8
	35.52	97.1	5.1	96.5	3.2	92.1	4.9	95.3	3.2	89.2	5.9
	177.60	90.8	7.7	93.5	5.1	91.2	3.7	92.5	4.8	85.9	8.7
Aztreonam	6.86	92.4	6.4	102.9	4.2	104.6	4.5	99.4	5.7	92.6	4.3
	34.32	96.5	3.8	96.5	3.8	99.3	5.7	95.7	4.4	90.6	5
	171.60	94.4	5.1	93.2	5.1	95.4	3.1	90.6	5.5	95	3.8
Meropenem	6.46	91.5	5.0	97.6	3.6	102.2	4.2	95.4	5.5	84.3	10.2
	32.32	96.2	4.3	98.1	3.2	104.3	3.4	96.2	4.4	75.1	9.6
	161.60	93.6	5.5	95.4	2.1	97.8	2.8	93.9	5.0	79.5	8.8
Imipenem	6.37	103.2	5.2	103.2	4.9	98.0	4.2	101.2	3.6	83.2	8.6
	31.84	94.7	6.9	97.3	4.5	95.7	3.1	95.8	5.3	77.5	5.9
	159.20	93.8	3.3	95.7	2.9	97.3	3.6	96.1	3.0	80.9	7.5
Levofloxacin	1.76	96.7	4.9	95.3	3.9	97.1	3.6	94.8	2.5	96.5	2.9
	8.82	97.2	3.5	98.3	4.6	102.2	4.2	97.3	2.0	93.9	3.5
	44.10	94.5	3.7	92.5	4.1	94.4	4.2	93.5	2.7	91.7	4.1
Moxifloxacin	0.91	90.6	6.7	103.3	2.1	104.1	3.4	104.4	3.1	96.3	4.3
	4.55	94.8	3.8	101.5	3.1	105.1	4.7	98.8	3.2	92.9	2.9
	22.75	93.9	3.2	97.4	2.8	95.7	3.2	95.4	4.3	93.4	5.1
Tigecycline	0.18	96.2	4.7	104.4	3.0	102.3	3.2	98.2	5.3	84.3	7.1
	0.92	93.9	2.9	102.1	4.0	105.6	3.7	92.9	4.4	81.4	6.2
	4.61	97.1	5.0	94.7	4.6	96.4	4.1	93.9	3.2	77.8	8.9
Azithromycin	0.69	92.7	5.1	105.6	2.8	103.3	2.5	101.4	4.5	93.2	4.5
	3.47	99.5	7.0	97.5	2.6	99.3	4.9	97.7	3.3	90.7	3.8
	17.36	94.3	3.6	93.6	3.7	95.3	2.2	93.5	3.4	89.5	6.7
Linezolid	1.23	96.3	3.3	100.8	4.7	98.1	3.0	103.8	4.0	90.5	4.5
	6.16	95.2	4.0	97.4	4.0	104.4	3.5	98.4	4.1	94.2	4.1
	30.80	91.8	4.9	94.6	4.3	96.3	4.3	93.5	3.3	92.6	5.2
Clindamycin	0.69	93.6	6.2	98.7	4.5	103.4	3.6	97.4	3.5	95.2	2.6
	3.45	97.2	4.9	101.3	5.2	105.9	3.8	99.7	4.1	97.6	3.3
	17.25	94.0	5.1	97.6	4.7	105.6	3.0	95.3	4.2	89.6	5.9
Voriconazole	0.33	91.6	3.8	99.1	3.5	98.4	3.6	94.4	4.6	89.7	5.2
	1.63	95.2	6.0	95.4	4.2	96.5	3.7	95.7	3.3	91.3	3.6
	8.16	98.4	4.3	92.7	4.3	93.5	4.2	90.4	4.0	93.9	4.1
Caspofungin	0.38	104.2	3.1	102.1	4.5	106.4	1.9	94.8	3.5	92.4	4.3
	1.92	95.4	4.4	97.5	3.3	101.3	2.4	96.2	2.9	89.7	6.9
	9.60	93.9	2.9	94.7	3.7	96.4	3.0	91.4	5.9	91.5	5.5
Vancomycin	1.30	89.9	5.7	95.4	2.9	96.3	3.0	93.6	5.5	81.8	8.2
	6.49	93.8	4.1	94.7	3.5	93.7	4.1	94.1	6.0	84.3	6.9
	32.44	94.2	3.8	92.2	2.0	95.4	2.3	91.5	4.4	79.7	9.8

#### 3.2.6 Dilution effects

These diluting QC samples were diluted 5-folds with blank plasma samples (*n* = 6). Precision (CV, %) was found between 3.5 and 6.9% while accuracy results were ranging from 94.3%–107.6% for all the antimicrobial agents. These results demonstrate that 5-fold dilution integrity is reliable for all antimicrobial agents, and the samples beyond calibration curves ranges can be determined accurately after the dilution.

### 3.3 Applicability of the method for routine TDM

In addition to the validation process, the developed LC-MS/MS method was successfully applied to determine the concentrations of 18 antimicrobial agents in 231 clinical samples obtained from Taihe Hospital. Hospital policies prevent the use of cefepime, itraconazole, and Posaconazole in our hospital. Patients without hepatic or renal impairment were chosen to avoid the influence on drug concentration. This study was approved by the Taihe Hospital Institutional Review Board and performed in compliance with the ethical standards of clinical research. Patients were administered intravenously, and plasma samples were obtained when reached steady-state concentrations after 5 to 7 times administration. Voriconazole and vancomycin levels were measured based on trough concentrations (blood was collected 30 min before the next administration), whereas the peak concentrations were measured for other drugs (blood was collected after intravenous administration). The dosages and concentrations of the 18 antimicrobial agents are summarized in Table 7. The results indicated that the concentrations of the antimicrobial agents were different in patients who had received the same dose, and the MIC of pathogenic bacteria in patients also differed. Therefore, the therapeutic effects also differed at the same dose. PK/PD studies can improve the therapeutic effect by combining the drug concentration and MIC of antibacterial drugs.

**TABLE 7:** Antimicrobial concentration ranges in 231 patient samples. (µg·mL^−1^).

Compounds	n	Dose	Concentration range	Median
Piperacillin	15	4.0 g, q 12 h	157.9–258.9	207.3
Cefazolin	15	1.5 g, q 12 h	93.7–193.3	139.5
Cefuroxime	11	1.5 g, q 12 h	68.9–101.2	77.9
Cefoperazone	13	2.0 g, q 8 h	133.6–221.0	174.3
Ceftriaxone	13	1.0 g, q12d	84.9–147.4	122.9
Cefepime	13	1.0g, q12 h	101.0–157.4	137.9
Aztreonam	7	1.0 g, q 12 h	79.9–120.8	103.2
Meropenem	12	1.0 g, q 8 h	30.6–61.4	47.7
Imipenem	14	1.0 g, q 8 h	38.9–71.2	55.0
Moxifloxacin	12	0.4 g, qd	2.8–6.3	4.8
Levofloxacin	12	0.5 g, qd	5.8–15.3	8.8
Tigecycline	13	50 mg, q12 h	0.5–1.2	0.8
Azithromycin	12	0.5 g, q 12 h	1.0–5.8	3.3
Linezolid	11	0.6 g,q 12 h	11.1–17.6	13.6
Clindamycin	12	0.6 g, q 12 h	3.5–10.2	6.4
Voriconazole	15	0.2 g, q 12 h	0.5–8.8	3.4
Caspofungin	15	50 mg, qd	1.6–8.1	4.7
Vancomycin	16	1.0 g, q 8 h	5.9–24.4	15.3

## 4 Conclusion

In this study, a sensitive and simple LC-MS/MS method was developed and validated for the simultaneous quantification of 18 antimicrobial agents in human plasma. Our method has significant advantages such as a low sample volume (50 µL) requirement and a short run time (6 min). The developed method was successfully validated for compliance with the guidelines for selectivity, linearity and LLOQ, precision and accuracy, matrix effect, extraction recovery, carryover, and stability. Lastly, this method was employed to quantify analytes in clinical samples from patients treated with antimicrobial agents.

## Data Availability

The raw data supporting the conclusion of this article will be made available by the authors, without undue reservation.

## Abbreviations

AMR, antimicrobial resistance; PK/PD, pharmacokinetic/pharmacodynamic; UPLC-MS, Ultra Performance Liquid Chromatography-tandem mass spectrometer; ESI, electrospray ionization; TDM, therapeutic drug monitoring; MIC, minimum inhibitory concentration; AUC, Area under the plasma concentration-time curve; IS, internal standard; QC, quality control; LLOQ, lower limit of quantification.

## References

[B1] Abdul-Aziz M. H. Alffenaar J. W. Bassetti M. Bracht H. Dimopoulos G. Marriott D. (2020). Antimicrobial therapeutic drug monitoring in critically ill adult patients: A position paper. Intensive Care Med. 46 (6), 1127–1153. 10.1007/s00134-020-06050-1 32383061 PMC7223855

[B2] Asín P. E. Rodríguez G. A. Isla A. (2015). Applications of the pharmacokinetic/pharmacodynamic (PK/PD) analysis of antimicrobial agents. J. Infect. Chemother. 21 (5), 319–329. 10.1016/j.jiac.2015.02.001 25737147

[B3] Aslam B. Khurshid M. Arshad M. I. Muzammil S. Rasool M. Yasmeen N. (2021). Antibiotic resistance: One health one World outlook. Front. Cell Infect. Microbiol. 11, 771510. 10.3389/fcimb.2021.771510 34900756 PMC8656695

[B4] Ates H. C. Roberts J. A. Lipman J. Cass A. E. Urban G. A. Dincer C. (2020). On-site therapeutic drug monitoring. Trends Biotechnol. 38 (11), 1262–1277. 10.1016/j.tibtech.2020.03.001 33058758

[B5] Chinese Pharmacopoeia Commission (2020). “Guidelines for validation of methods for quantitative analysis of biological samples,” in Pharmacopoeia of the people’s republic of China. Beijing, China Medical Science Press, 466–472.

[B6] Couet W. (2018). Pharmacokinetics/pharmacodynamics characterization of combined antimicrobial agents: A real challenge and an urgent need. Clin. Microbiol. Infect. 24 (7), 687–688. 10.1016/j.cmi.2018.03.047 29649606

[B7] FDA (2018). Bioanalytical method validation guidance for industry. Available at: https://www.fda.gov/regulatory-information/search-fda-guidance-documents/bioanalytical-method-validation-guidance-industry .

[B8] Ferrari D. Ripa M. Premaschi S. Banfi G. Castagna A. Locatelli M. (2019). LC-MS/MS method for simultaneous determination of linezolid, meropenem, piperacillin and teicoplanin in human plasma samples. J. Pharm. Biomed. Anal. 169, 11–18. 10.1016/j.jpba.2019.02.037 30826487

[B9] Hahn J. Choi J. H. Chang M. J. (2017). Pharmacokinetic changes of antibiotic, antiviral, antituberculosis and antifungal agents during extracorporeal membrane oxygenation in critically ill adult patients. J. Clin. Pharm. Ther. 42 (6), 661–671. 10.1111/jcpt.12636 28948652

[B10] Huemer M. Shambat S. M. Brugger S. D. Zinkernagel A. S. (2020). Antibiotic resistance and persistence -Implications for human health and treatment perspectives. EMBO Rep. 21(12). e51034. doi: 10.15252/embr.202051034 33400359 PMC7726816

[B11] Kang J. S. Lee M. H. (2009). Overview of therapeutic drug monitoring. Korean J. Intern Med. 24 (1), 1–10. 10.3904/kjim.2009.24.1.1 19270474 PMC2687654

[B12] Klein E. Y. Milkowska-Shibata M. Tseng K. K. Sharland M. Gandra S. Pulcini C. (2021). Assessment of WHO antibiotic consumption and access targets in 76 countries, 2000-15: An analysis of pharmaceutical sales data. Lancet Infect. Dis. 21 (1), 107–115. 10.1016/S1473-3099(20)30332-7 32717205

[B13] Lepak A. J. Andes D. R. (2014). Antifungal pharmacokinetics and pharmacodynamics. Cold Spring Harb. Perspect. Med. 5 (5), a019653. 10.1101/cshperspect.a019653 25384765 PMC4448584

[B14] Luo X. X. Xue X. C. Li T. F. Zhang Y. Huang L. Cheng G. (2020). Differential impacts of azole antifungal drugs on the pharmacokinetic profiles of dasatinib in rats by LC-MS-MS. Curr. Drug Metab. 21 (13), 1022–1030. 10.2174/1389200221666201022140656 33092505

[B15] Matuszewski B. K. Constanzer M. L. Chavez-Eng C. M. (2003). Strategies for the assessment of matrix effect in quantitative bioanalytical methods based on HPLC-MS/MS. Anal. Chem. 75, 3019–3030. 10.1021/ac020361s 12964746

[B16] Moreheadn M. S. Scarbrough C. (2018). Emergence of global antibiotic resistance. Prim. Care 45 (3), 467–484. 10.1016/j.pop.2018.05.006 30115335

[B17] Mouton J. W. Dudley M. N. Cars O. Derendorf H. Drusano G. L. (2005). Standardization of pharmacokinetic/pharmacodynamic (PK/PD) terminology for anti-infective drugs: An update. J. Antimicrob. Chemother. 55 (5), 601–607. 10.1093/jac/dki079 15772142

[B18] Nellums L. B. Thompson H. Holmes A. Castro-Sánchez E. Otter J. A. Norredam M. (2018). Antimicrobial resistance among migrants in europe: A systematic review and meta-analysis. Lancet Infect. Dis. 18 (7), 796–811. 10.1016/S1473-3099(18)30219-6 29779917 PMC6032478

[B19] Parker S. L. Valero Y. C. Mejia J. L. Roger C. Lipman J. Roberts J. (2017). An LC-MS/MS method to determine vancomycin in plasma (total and unbound), urine and renal replacement therapy effluent. Bioanalysis 9 (12), 911–924. 10.4155/bio-2017-0019 28617036

[B20] Patel B. Suhagiac B. N. Jangidb A. G. Mistrib H. N. Desaia N. (2016). Systematic evaluation of matrix effect and cross-talk-free method for simultaneous determination of zolmitriptan and N-desmethyl zolmitriptan in human plasma: A sensitive LC-MS/MS method validation and its application to a clinical pharmacokinetic study. Biomed. Chromatogr. 30 (3), 447–458. 10.1002/bmc.3568 26189757

[B21] Rehm S. Rentsch K. M. (2020b). HILIC LC-MS/MS method for the quantification of cefepime, imipenem and meropenem. J. Pharm. Biomed. Anal. 186, 113289. 10.1016/j.jpba.2020.113289 32428767

[B22] Rehm S. Rentsch K. M. (2020a). LC-MS/MS method for nine different antibiotics. Clin. Chim. Acta 511, 360–367. 10.1016/j.cca.2020.11.001 33159947

[B23] Rochford C. Sridhar D. Woods N. Saleh Z. Hartenstein L. Ahlawat H. (2018). Global governance of antimicrobial resistance. Lancet 391 (10134), 1976–1978. 10.1016/S0140-6736(18)31117-6 29864011

[B24] Rodríguez G. A. Solinís M. Á. Isla A. (2021). The role of PK/PD analysis in the development and evaluation of antimicrobials. Pharmaceutics 13 (6), 833. 10.3390/pharmaceutics13060833 34205113 PMC8230268

[B25] Scaglione F. Paraboni L. (2008). Pharmacokinetics/pharmacodynamics of antibacterials in the intensive care unit: Setting appropriate dosing regimens. Int. J. Antimicrob. Agents 32 (4), 294–301. 10.1016/j.ijantimicag.2008.03.015 18621508

[B26] Sinnollareddy M. G. Roberts M. S. Lipman J. Roberts J. A. (2012). β-Lactam pharmacokinetics and pharmacodynamics in critically ill patients and strategies for dose optimization: A structured review. Clin. Exp. Pharmacol. Physiol. 39 (6), 489–496. 10.1111/j.1440-1681.2012.05715.x 22519600

[B27] Veiga R. P. Paiva J. A. (2018). Pharmacokinetics-pharmacodynamics issues relevant for the clinical use of beta-lactam antibiotics in critically ill patients. Crit. Care 22 (1), 233. 10.1186/s13054-018-2155-1 30244674 PMC6151903

[B28] Williams P. Cotta M. O. Roberts J. A. (2019). Pharmacokinetics/Pharmacodynamics of β-lactams and therapeutic drug monitoring: From theory to practical issues in the intensive care unit. Semin. Respir. Crit. Care Med. 40 (4), 476–487. 10.1055/s-0039-1693498 31585474

[B29] Zhang C. N. Ma W. D. Zhang Y. H. Wang Q. B. Chen L. Zheng T. (2018). Pharmacokinetics, bioavailability, and tissue distribution study of angoroside C and its metabolite ferulic acid in rat using UPLC-MS/MS. Front. Pharmacol. 23 (9), 1186. 10.3389/fphar.2018.01186 PMC6206173 30405411

